# Juxta-Articular Osteoid Osteoma With Inflammatory Femoroacetabular Impingement: A Case Report

**DOI:** 10.7759/cureus.79946

**Published:** 2025-03-03

**Authors:** Dai Otsuki, Chikahisa Higuchi, Daisuke Tamura, Haruka Guda, Mai Konishi

**Affiliations:** 1 Department of Orthopedic Surgery, Osaka Women's and Children's Hospital, Izumi, JPN; 2 Department of Rehabilitation, Osaka Women's and Children's Hospital, Izumi, JPN; 3 Department of Orthopedic Surgery, Osaka University Graduate School of Medicine, Suita, JPN

**Keywords:** femoroacetabular impingement, hip arthritis, inflammatory femoroacetabular impingement, juxta-articular, osteoid osteoma

## Abstract

Inflammatory femoroacetabular impingement (FAI) is a cam lesion caused by inflammation and is characterized by a distinct bone deformity. To date, no reports have described bone deformities resulting from juxta-articular osteoid osteomas that resemble those seen in inflammatory FAI. We report a case of a 15-year-old girl who presented with right hip pain, hip arthritis, and a cam deformity at the femoral head-neck junction. Computed tomography revealed a bone translucency with internal calcification, suggestive of a nidus, at the medial cortex of the right femoral neck. Based on these findings, we diagnosed inflammatory FAI caused by juxta-articular osteoid osteoma. Following resection, her pain resolved rapidly. This is a rare reported case of juxta-articular osteoid osteoma causing inflammatory FAI. Understanding the characteristics of inflammatory FAI can aid in distinguishing between inflammation and FAI as the source of pain.

## Introduction

Osteoid osteoma is a benign osteogenic tumor that most commonly affects the femur and tibia in adolescents and young adults in their teens and 20s [1]. It is well known for causing nocturnal pain, which responds remarkably well to analgesics [2,3]. Compared to extra-articular osteoid osteoma, intra-articular or juxta-articular osteoid osteomas are less likely to present with characteristic nocturnal pain, often leading to a delayed diagnosis [4,5]. In particular, intra-articular or juxta-articular osteoid osteomas frequently manifest with arthritis and may be associated with synovitis and bone deformities [6,7]. Therefore, differentiation from other conditions, such as juvenile idiopathic arthritis and osteomyelitis, is essential [8].

Bone deformities in the hip joint can lead to femoroacetabular impingement (FAI). FAI is classified into three types based on bone morphology: pincer, cam, and mixed types [9,10]. Pincer-type FAI is characterized by excessive coverage of the anterior-superior rim of the acetabulum, with the cross-over sign being a well-known radiographic feature. Cam-type FAI is defined by a cartilage or bony prominence at the femoral head-neck junction, resulting in an aspherical femoral head. Mixed-type FAI exhibits characteristics of both cam-type and pincer-type FAI.

In 2020, inflammatory FAI, characterized by distinctive cam lesions thought to be caused by arthritis, was proposed by Seth et al. [11]. Inflammatory FAI refers to cam deformity of the femoral head resulting from arthritis, leading to a cam lesion with a large, sharp bump, a negative head-neck offset, and an increased alpha angle. In their report, the authors identified inflammatory conditions associated with inflammatory FAI, including ankylosing spondylitis, Crohn's disease, juvenile idiopathic arthritis, and Hashimoto's thyroiditis. However, there have been only a few reports of inflammatory FAI caused by osteoid osteoma [12,13]. This report describes a case of inflammatory FAI induced by juxta-articular osteoid osteoma-associated arthritis.

## Case presentation

**Figure 1:**
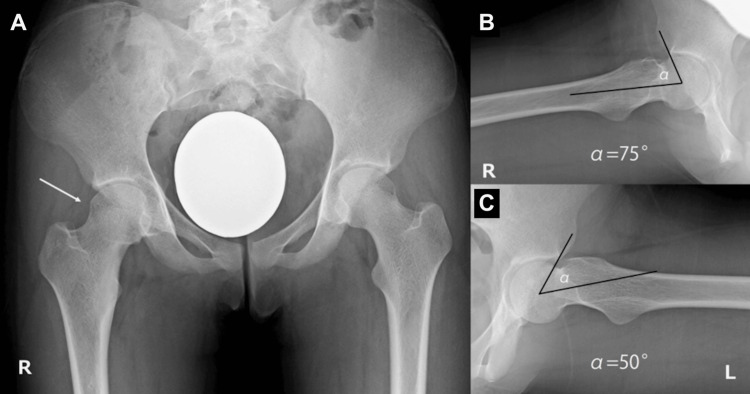
Preoperative anteroposterior (AP) and lateral radiograph of the hip joint (A) Bilateral cross-over signs and a sharp bony bump in the right femoral head-neck junction (arrow). (B) The alpha angles were 75° at the right hip and (C) 50° at the left hip.

A 15-year-old girl presented to our clinic with chief complaints of right hip pain and difficulty sitting in deep hip flexion. At rest, her hip pain was not severe enough to require analgesics at night. She was able to walk but experienced pain during physical activity. On examination, tenderness was noted in the right hip, and external rotation of the hip joint was observed during flexion, resembling a Drehmann sign. The range of motion of the right hip joint was restricted, with flexion limited to 100° and internal rotation to -10°. Radiographic evaluation revealed a prominent, sharply defined bony bump at the head-neck junction of the right femur and a cross-over sign on the acetabular side (Figure 1).

The alpha angle of the right hip was increased to 75° compared to 50° on the left hip. No increase in the head-neck offset was observed.

**Figure 2:**
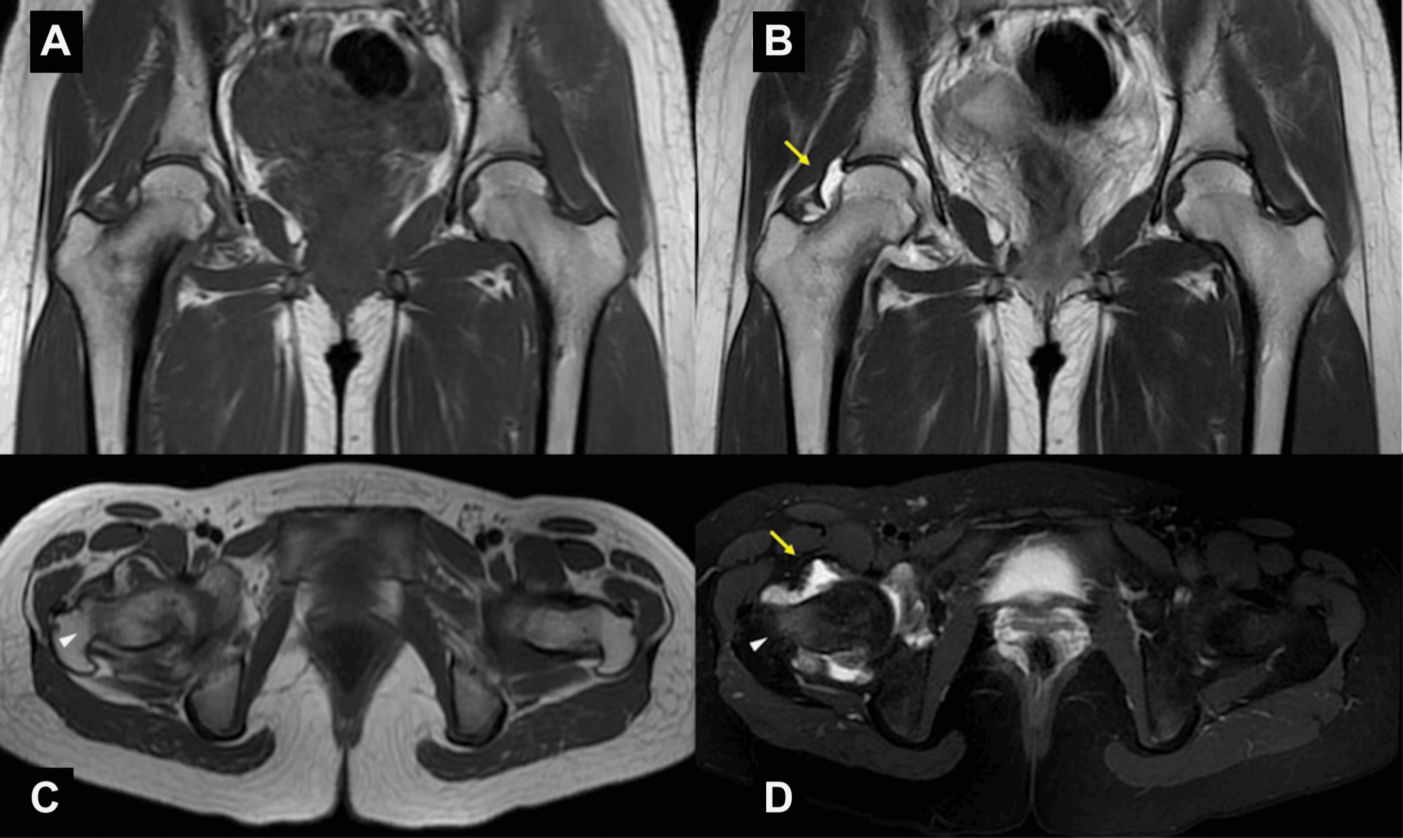
Preoperative magnetic resonance imaging (MRI) T1-weighted coronal/axial (A, C), T2-weighted coronal (B), T2-weighted fat-suppression axial (D). The yellow arrows show the joint effusion. The white arrowheads show the bone marrow edema at the femoral neck.

Magnetic resonance imaging (MRI) revealed high signal intensity on T2-weighted images and low signal intensity on T1-weighted images in the right hip joint. Additionally, the bone marrow of the medial femoral neck exhibited high signal intensity on T2-weighted fat-suppressed images and low signal intensity on T1-weighted images. These findings suggested the presence of fluid retention with synovitis in the right hip joint, accompanied by bone marrow edema (Figure 2).

**Figure 3:**
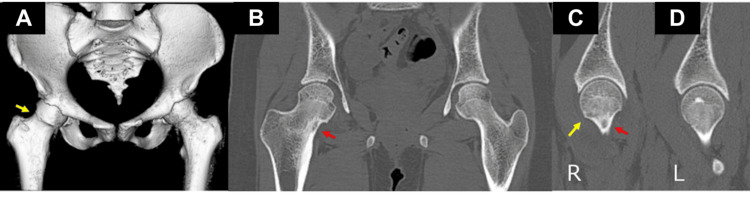
Preoperative computed tomography (CT) images The yellow arrow shows the bony bump on three-dimensional CT (A) and sagittal CT images (C). There was no bony bump on sagittal CT images (D). The red arrows show a nidus (B, C).

Computed tomography (CT) imaging demonstrated a radiolucent lesion with internal calcification, suspected to be a nidus, located in the medial cortex of the right femoral neck (Figure 3).

Based on these findings, we diagnosed arthritis secondary to juxta-articular osteoid osteoma in the right hip joint rather than arthritis due to FAI.

We opted for a minimally invasive approach, avoiding direct hip joint exposure. Instead, we created a bony foramen in the lateral cortex of the femur and resected the tumor through this access point. The procedure was performed under general anesthesia with the patient in the supine position. Using a lateral approach to the right femur, a Kirschner wire was inserted from the lateral cortex toward the tumor, with the tumor's location confirmed using fluoroscopy in conjunction with preoperative CT imaging. The tumor was subsequently curetted, and endoscopic visualization was used to confirm complete resection. Finally, the drilled cavity was filled with beta-tricalcium phosphate artificial bone (Osferion®, Olympus, Tokyo, Japan) to promote bone regeneration.

**Figure 4:**
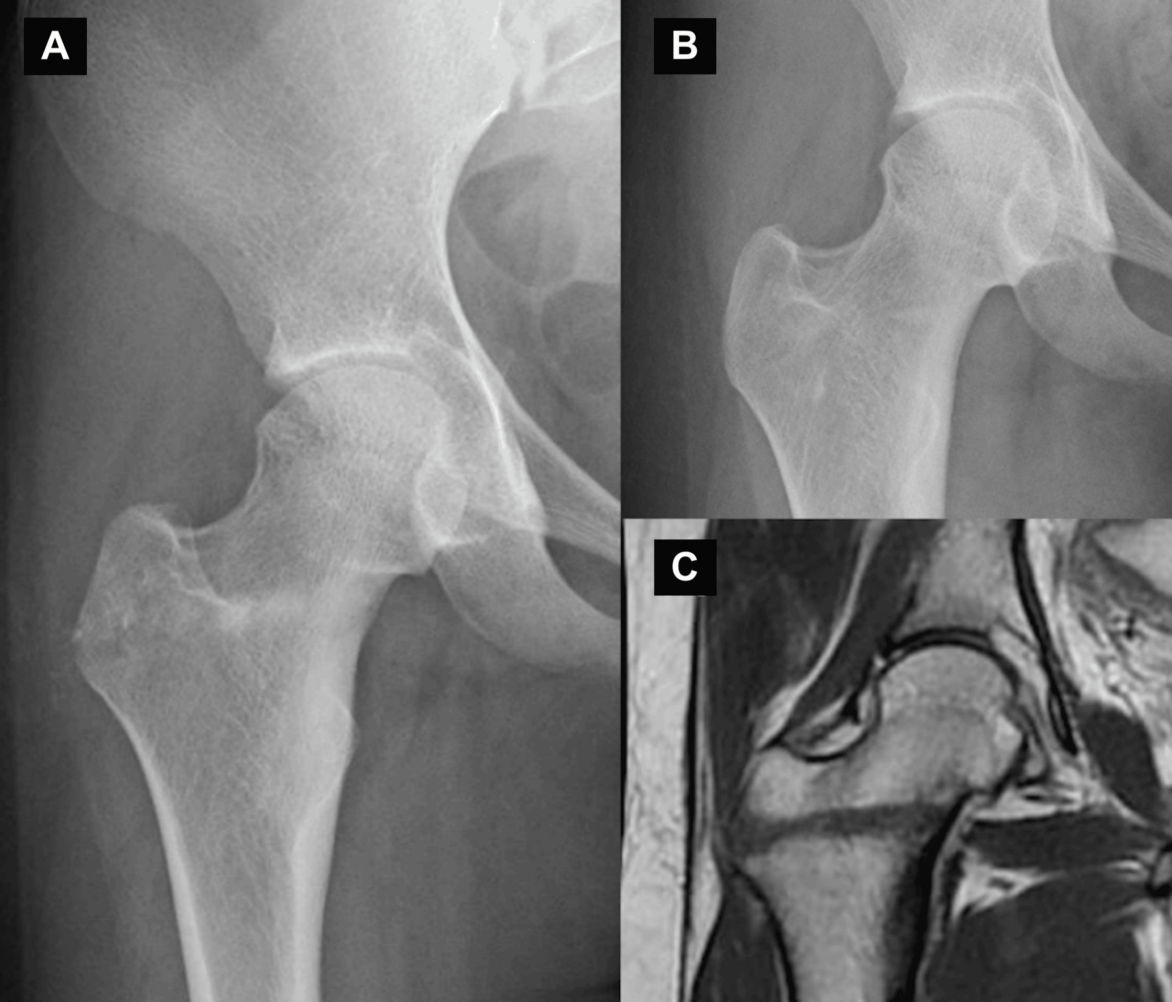
Postoperative images (A) Immediately after the operation; (B) nine months after the operation. The bony bump remains at the femoral head-neck junction. (C) T2-weighed magnetic resonance imaging shows the disappearance of the high signal intensity in the right hip joint.

The histopathological diagnosis confirmed osteoid osteoma. Postoperatively, the pain resolved immediately, and an MRI performed nine months after surgery showed complete resolution of the hip arthritis (Figure 4).

Two years after surgery, the pain has remained absent; however, a cam lesion persists in the right femoral neck.

## Discussion

Osteoid osteoma is most commonly found in the diaphysis of the femur and tibia [2], but it can also occur near or within the hip joint. The nidus of osteoid osteoma produces prostaglandins, particularly prostaglandin E2, which induces characteristic nocturnal pain due to inflammation [14,15].

Song et al. reported on the clinical and radiological features of intra-articular and extra-articular osteoid osteomas [4]. Their study showed nocturnal pain in 70% of extra-articular osteoid osteomas, compared to only 36% of intra- and juxta-articular cases. Intra-articular synovitis was absent in extra-articular osteoid osteomas but present in 72% of intra- and juxta-articular cases. Additionally, bone deformation secondary to inflammation was observed in only 4% of extra-articular osteoid osteomas but in 45% of intra- and juxta-articular cases. As a result, the diagnosis of intra-articular and periarticular osteoid osteomas is often delayed due to the absence of typical symptoms and imaging findings. The time to diagnosis for intra- and juxta-articular osteoid osteomas was significantly delayed, averaging 12 months, compared to only 1.5 months for extra-articular cases. Intra- and juxta-articular osteoid osteomas are frequently misdiagnosed as chronic osteomyelitis, sprains, or pigmented villonodular synovitis. Since these cases can present with arthritis-like symptoms, it is essential to differentiate them from osteomyelitis, juvenile idiopathic arthritis, and chronic recurrent multifocal osteomyelitis [8].

Cam-type FAI is common among athletes and is associated with sports that require a large range of motion in the hip joint [10,16]. In contrast, inflammatory FAI, in which inflammation induces cam-type FAI, was first reported in 2020 [11]. Inflammatory FAI is characterized by a large, sharp bump, a negative head-neck offset, and a large alpha angle.

In our case, the initial diagnosis was mixed-type FAI due to the loss of roundness at the femoral head-neck junction (cam deformity) and a positive cross-over sign (pincer deformity). However, MRI revealed synovial hyperplasia and significant bone marrow edema in the hip joint, findings inconsistent with pain caused by FAI. A CT scan identified a nidus near the hip joint, and the femoral cam lesion exhibited characteristics consistent with inflammatory FAI (a large, sharp bump, a negative head-neck offset, and a large alpha angle). Therefore, we diagnosed inflammatory FAI caused by juxta-articular osteoid osteoma.

After discussing the treatment plan with the patient, we opted for tumor excision alone, without direct treatment for FAI, to alleviate hip pain. The pain resolved rapidly after surgery.

There is one reported case of cam-type FAI caused by juxta-articular osteoid osteoma [17]. In that case, synovitis due to juxta-articular osteoid osteoma was observed. However, since the patient was an ice hockey player, it was necessary to determine whether the cam deformity resulted from sports activity or inflammation caused by the osteoid osteoma. The specific characteristics of the cam-type lesion were not described in the report. When pain is caused by a cam deformity, shaving the cam lesion is necessary. In contrast, if inflammation due to juxta-articular osteoid osteoma is the source of pain, resection of the osteoid osteoma is required. Differentiating cam-type FAI from inflammatory FAI is crucial for determining the appropriate treatment strategy.

## Conclusions

We presented a case of inflammatory cam deformity caused by osteoid osteoma. It is essential to determine whether the pain originates from cam-type FAI or inflammation. Understanding the characteristics of femoral head deformities in inflammatory FAI can aid in making this distinction.
